# Organized Sport Participation, Physical Activity, Sleep and Screen Time in 16-Year-Old Adolescents

**DOI:** 10.3390/ijerph18063162

**Published:** 2021-03-18

**Authors:** Elvar S. Saevarsson, Vaka Rognvaldsdottir, Runa Stefansdottir, Erlingur Johannsson

**Affiliations:** 1 Faculty of Health Promotion, Sport Sciences and Leisure Studies, School of Education, University of Iceland, 105 Reykjavík, Iceland; elvarss@gmail.com (E.S.S.); rss7@hi.is (R.S.); erljo@hi.is (E.J.); 2 Department of Sport and Physical Activity, Western Norway University of Applied Sciences, 5063 Bergen, Norway

**Keywords:** organized sport participation, adolescents, physical activity, screen time, sedentary behavior, body composition

## Abstract

This study aimed to examine the association of different frequencies of organized sport participation (OSP) with physical activity, cardiorespiratory fitness, body composition, sleep, and screen time among adolescents. A cross-sectional study involving 315 16-year-old adolescents was conducted. OSP was self-reported, being categorized as 0 times a week, less than three times a week, 4–5 times a week, and 6–7 times a week, on average. Screen time was also self-reported but physical activity and sleep duration were objectively measured. Cardiorespiratory fitness and body composition were measured using a maximal cycle ergometer test and a dual-energy X-ray absorptiometry (DXA) scan, respectively. An analysis of covariance revealed a significant association between OSP and physical activity (F (3, 286) = 14.53, *p* < 0.01), cardiorespiratory fitness (F (3, 236) = 17.64, *p* < 0.01), screen time (F (3, 294) = 8.14, *p* < 0.01), body fat percentage (F (3, 292) = 11.84, *p* < 0.01), and fat free mass (F (3, 290) = 5.76, *p* < 0.01. No significant association was found between OSP and sleep duration. Post hoc analyses showed that OSP at least four times a week was beneficial to favorable physical activity levels, cardiorespiratory fitness, screen time, and body composition and may therefore serve as a valuable tool in battling unhealthy lifestyle behaviors among adolescents.

## 1. Introduction

Unhealthy behaviors including low physical activity levels comprise one of the key health challenges facing European adolescents [1]. Although physical activity has been found to benefit both physical and mental health in children and adolescents, more than eight out of ten students aged 11–17 years globally [2,3], and six out of ten students in Iceland [4] did not meet the recommended daily 60 min of moderate to vigorous-intensity physical activity in 2016 and 2015, respectively. Sedentary behavior has also been deleteriously associated with numerous health indicators in children and youth including quality of life, self-esteem, depression, and anxiety [5]. These unhealthy lifestyle behaviors have led to a rise in childhood adiposity in the last few decades, leading to approximately one in every six children being considered overweight or obese [6] which can have a detrimental effect on cardiovascular risk factors [7], mental health [8], type 2 diabetes and several cancers in adult life [9], and may result in greater victimization and exclusion [10]. Sleep can also be viewed as a health behavior since insufficient sleep has been associated with several negative health markers, including adiposity and mental health outcomes [11]. Research has shown that short sleep duration is common among children and adolescents and sleep duration has declined over the past decades [12,13,14]. About 30%–70% of European and American children have poor sleep patterns [15] and only 30% of American adolescents achieve 8–10 h nightly sleep on school days [16,17]. Insufficient sleep has been reported previously in Icelandic adolescents, with reported average sleep time on school days being only 6.2 h, which is well below the recommended 8 to 10 h [18], revealing a substantial sleep deficiency [19].

Organized sport participation (OSP) is offered to children and adolescents in most Western and non-Western countries, with about two thirds of children being actively engaged in Europe [20]. Icelandic data show that participation rates have increased among 14 and 15-year-olds, from 40% in 1992 to 60% in 2014, with the highest participation rates among 12-year-olds, 83% [21]. OSP has been found to be positively associated with increased physical activity levels among participants [22,23] and may therefore be a good tool in the battle against low physical activity levels among children and adolescence. However, its associations with sedentary behavior, e.g., screen time use, and sleep are inconclusive and need to be studied in more detail. Most studies on the topic have dichotomized OSP as participants vs. non-participants, which might underestimate the impact of intense OSP on the above-mentioned markers of health. In support, in our previous work, a threshold effect in the association between OSP and performance in math was detected [24].

The purpose of this study was therefore to explore the relationship between OSP, physical activity, sleep, sedentary behavior, and adiposity by categorizing participants into four groups based on their weekly frequency of participation. This allows for the detection of possible dose-response relationships or the threshold effect of ‘how much is enough’.

## 2. Materials and Methods

### 2.1. Sample and Data Collection

The data used in this study stems from an Icelandic public health study named Lifestyle of 7–9-Year-Old Children conducted between 2006 and 2008 in six randomly selected schools in Reykjavik [25]. Participants from that study were given the option to participate in a follow-up study in 2015, when they were in the tenth grade, named Health Behavior of Icelandic Youth. In April, a total of 411 adolescents (58% girls) were approached and 315 (53% girls) accepted the offer. Non-participation (n = 96) was mainly due to absence from school during measurement days and lack of interest in the study. The study took place during school time from April-June. Students who did not attend school (sick or travelling) during days of measurements at individual schools were excluded. Those who became ill within the week of data collection (while wearing the accelerometer) were not excluded nor registered. Written informed consent was obtained from all participants and their guardians. Strict procedures were followed to ensure confidentiality.

### 2.2. Objectively Measured Physical Activity and Sleep

Free-living physical activity and sleep parameters were objectively measured using triaxial raw signal accelerometer based ActiGraph activity monitors (model GT3X+ ActiSleep, ActiGraph Inc., Pensacola, FL, USA). Raw triaxial data (in milliG) sampled at 80 samples per second (Hz) were reduced to the vector magnitude of activity counts over 60 s epochs and averaged over all valid days using ActiLife software from ActiGraph (Pensacola, FL, USA; version 6.13.0) and customized programs in MATLAB (the Mathworks, Natick, MA, USA; version R2013a). Sleep parameters were calculated using the Sadeh sleep detection algorithm specifically validated for adolescents [26]. Wrist-actigraphy has high sensitivity and moderate specificity and overall high accuracy when compared to polysomnography [27]. It has been recommended for the characterization of sleep parameters in population-based studies of young adults [28]. Each participant was asked to continuously wear the monitor on the non-dominant wrist for seven consecutive days. Since the model GT3X+ is water resistant, the subjects should have worn the monitor during water-based activities. A minimum of three valid school days and one valid non-school day was set as an inclusion criterion for the study. Days with a wear time of ≥ 14 h from 0:00 to 23:59:59 were considered valid for the study. The wear time criterion is in line with recent systematic review of 10 h/day wear time, although studies assessing 24 h continuous activity and sleep may require longer wear times than studies focusing on daytime activity only [29]. Physical activity was presented as total physical activity (average counts per minute of wear time—ctm) and sleep as total sleep duration in minutes.

### 2.3. Cardiorespiratory Fitness

Cardiorespiratory fitness (CRF) was measured with a graded maximal exercise test, using a Monark 829E electronically braked cycle ergometer (Monark Exercise AB, Vansbro, Sweden). Boys started out with a 50 W resistance, which was increased by another 50 W every three minutes until exhaustion. Girls started out with a 40 W resistance, increased by another 40 W every three minutes until exhaustion [30]. The recommended pedaling rate was 70 rpm and the tests were terminated if the rate fell below 40 rpm. Heart rate was measured with a Polar heart rate monitor via chest band (Polar Vantage) as well as ratings of perceived exertion at the end of each level. The test was considered maximal if at least two out of the three following criteria were met: a heart rate no more than 5% below the age-predicted maximum, calculated as (207 − (0.7 × age) ± 10 beats), a score of 19–20 on the Borg scale of perceived exertion, and the researchers’ subjective estimates of maximal effort. CRF, expressed as maximal power output with linear regression scaling, was used in final analyses with fat free mass (FFM) entered as a covariate.

### 2.4. Anthropometry

Anthropometric measurements were conducted at participants’ schools. Standing height was measured with a stadiometer (Seca model 217, Seca Ltd. Birmingham, UK) to the nearest 0.1 cm. Body weight was measured to the nearest 0.1 kg using a scale (Seca model 813, Seca Ltd. Birmingham, UK) with participants wearing light clothes. This allowed for the calculation of body mass index (BMI: kg/m^2^). In addition, body composition was measured with dual-energy X-ray absorptiometry (DXA) using a GE LUNAR iDXA scanner (General Electric Lunar, Wauwatosa, WI, USA) at the Icelandic Heart Association. All DXA measurements were performed by a certified radiologist.

### 2.5. Organized Sport Participation and Screen Time

Participants completed a questionnaire assessing various health-related behaviors. OSP was assessed by the question: ‘How often do you participate (practice or compete) in sports with a club per week?’ The response options were: ‘Never’ (group 1); ‘Less than once a week’, ‘Once a week’, ‘2–3 times a week’ (group 2); ‘4–5 times a week’ (group 3); and ‘Almost every day’ (group 4). Due to the low response rate (total 12 for first two options) and because no statistically significant mean difference on key variables was detected in ‘Less than once a week’ and ‘Once a week’, those options were merged with ‘2–3 times a week’ to represent all those who reported being engaged in organized sports three times a week or less (group 2). The ‘Almost every day’ option was renamed ‘6–7 times a week’ in this study.

Participants were asked to report the number of hours per day on average, separately for school and non-school days, they spent playing computer games, watching TV/DVD/internet material, using the internet for web-browsing/Facebook/e-mail, and participating in ‘other’ computer use. Each item was scored on a seven-point Likert scale, with the following response options: 1 = ‘none’, 2 = ‘about ½ h’, 3 = ‘1 to 2 h’, 4 = ‘2 to 3 h’, 5 = ‘3 to 4 h’, 6 = ‘4 to 5 h’ and 7 = ‘more than 5 h’. Average daily hours for each type of screen-based activity were computed, using the midpoints for scoring categories and weighted averages for school and non-school days. All screen-based activities were then summed to arrive at the total daily screen time (h/day).

Parental education was used to assess socioeconomic status (dichotomous variable, one or both parents with a university degree = 1, neither parent with a university degree = 0). No significant difference was detected between the two groups on any of the study variables, so parental education was not included in the analyses.

### 2.6. Statistics

Statistical analyses were conducted with SPSS 26.0 statistical package (IBM SPSS Statistics for Windows, Version 26.0. Armonk, NY, USA: IBM Corp). Variables were inspected for normality and logarithmic transformations performed on total activity, body weight, FFM, and total screen time to correct for skewness. The untransformed values are displayed in Table 1. Independent t-tests and Mann-Whitney U tests were used to assess the mean differences between the sexes. Chi squared tests with Bonferroni correction for multiple comparisons were used to assess sex difference in the weekly frequency of OSP [31]. The relationship between independent and dependent variables and the inspection of the groups’ mean differences were assessed using analyses of covariance (ANCOVA) with the groups’ estimated marginal means displayed. The interaction between OSP and sex were tested in all models and, if statistically significant, the analyses were stratified by sex (in total physical activity only). Levine’s test for equal group variances and White’s test for heteroscedasticity were performed in all analyses. Results using different methods to adjust CRF for different body sizes (FFM, FFM^2/3^ and linear regression scaling) were compared but did not vary. Statistical significance was accepted at an alpha level of 0.05.

## 3. Results

Participants’ characteristics are presented in Table 1. Boys were significantly taller and heavier than the girls and had more FFM and better CRF. The boys had significantly more daily screen time, about 37 min, than the girls. Girls had significantly higher body fat percentage than boys. No sex differences were detected in total physical activity or age.

**Figure 1:**
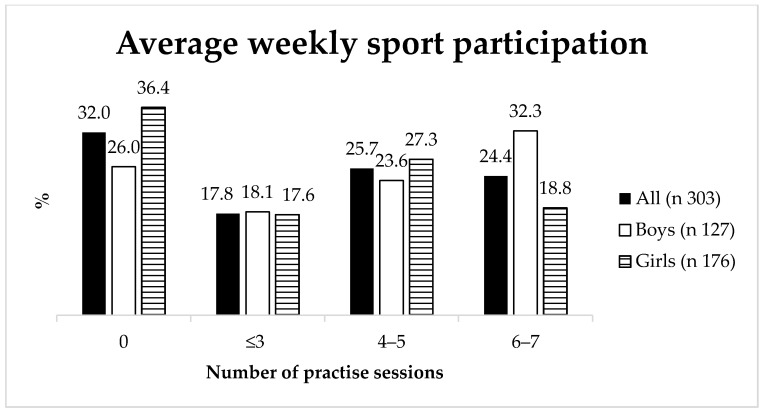
Number of practice sessions per week in total and by sex.

Responses to the question ‘How often do you participate (practice or compete) in sports with a club per week?’ are displayed in Figure 1. Almost 60% of the respondents indicated that they were actively engaged in organized sports and almost 25% responded that they do so on most days of the week. A Chi squared test revealed a significant sex difference in favor of boys in weekly OSP frequency χ^2^ (3, *N* = 303) = 8.41, *p* = 0.04, which was rendered insignificant after the Bonferroni post hoc adjustment for multiple comparisons (*p* > 0.0063).

Results from the ANCOVA between OSP, health behavior, and body composition are displayed in Table 2. A significant positive association between OSP and total physical activity was detected for not only the whole sample but also for each sex separately. Furthermore, OSP was positively significantly associated with CRF, even after further adjustments for total physical activity. There was a significant negative association between OSP and screen time. OSP was associated significantly with both body composition variables, negatively with body fat percentage, and positively with FFM. The association between OSP and body fat percentage was still significant after further adjustment for total physical activity. However, no significant association between OSP and sleep was found.

Results from ANCOVA post hoc analyses are displayed in Table 3 showing the differences in OSP frequency groups estimated means. The most active groups, groups 3 and 4, had significantly higher total physical activity than the two less active groups, groups 1 and 2. Among boys, groups 3 and 4 had significantly higher total physical activity than did groups 1 and 2, but among girls a significant difference was only detected between group 4 and group 1. Groups 3 and 4 had significantly better CRF than groups 1 and 2. Further adjustments for total physical activity did not impact the association. Indications are for the dose dependent association between OSP and CRF. No significant difference between the groups was detected with regard to sleep duration. Screen time was significantly lower in groups 3 and 4 than in groups 1 and 2. Adiposity levels were significantly lower in group 3 than in group 1, and group 4 had lower levels than both groups 1 and 2. Further adjustment for physical activity did not have an effect on the association. FFM levels were significantly higher in group 4 than in groups 1 and 2. No difference in BMI was detected between the groups (data not shown).

## 4. Discussion

The main findings of this study suggest a favorable association of OSP with total physical activity, screen time, and body composition. The findings show that for OSP to significantly impact total physical activity, CRF, body fat percentage, and screen time, it is necessary to participate at least four times a week. Additionally, more frequent participation, at least six times a week, will result in more FFM. Those who reported practicing three times a week or less did not differ from those who did not engage in OSP. These findings support our speculations that dichotomizing OSP may underestimate the effect more frequent participation may have on health behavior and body composition. No significant relationship between OSP and objectively measured sleep was detected.

### 4.1. Physical Activity

The current findings of a positive association between OSP and total physical activity correspond to previous studies. Marques et al. (2016) reported that, among 10–18-year-olds, those who were engaged in organized sports were more likely to achieve the recommended amount of physical activity than were those who did not participate [22]. Hebert et al. (2015) reported higher levels of physical activity with more frequent OSP in four out of five sports explored, showing the association to be dose dependent [23]. In the current study, there are indications for a dose-dependent association that supports a causal association. The contribution of OSP to total physical activity may have been underestimated in previous studies that dichotomize OSP. The findings in this study reject the ActivityStat hypothesis since there does not seem to be a compensatory decrease in other physical activity domains to maintain an overall stable level of physical activity over time.

### 4.2. Cardiorespiratory Fitness

This study’s findings of a positive association between OSP and CRF, independent of total physical activity, correspond to those of previous studies. Drenowatz et al. (2019) reported better performance in a 6 min run test among those engaged in OSP in 6–14-year-old children [32]. Carlisle et al. (2019) found that those who reported to be engaged in OSP performed better in the Cooper 12 min run/walk test than did those who were not engaged in OSP [33]. These studies did, however, dichotomize OSP thereby eliminating all indications of a causal association. The current findings show a significant threshold effect: participating in organized sport at least four times a week is necessary to impact CRF levels. The association between OSP and CRF seems to be dose dependent, which again indicates a causal relationship. CRF reflects the level of moderate-to-vigorous physical activity (MVPA) of the foregoing weeks or months and may be used as a proxy for those activities [34]. Therefore, it seems that OSP involves enough MVPA to impact CRF among participants. However, selection bias cannot be ruled out since 40% of CRF is attributable to genetics [34].

### 4.3. Body Composition

The current findings show a significant favorable association between OSP and adiposity. Participating in organized sport four times a week or more resulted in significantly lower adiposity levels compared to the less active groups. In the existing literature, the association between OSP and body fat percentage is inconclusive, although Telford et al. (2015) reported a negative association between OSP and body fat percentage measured by DXA only in 8–16-year-old girls [35]. Vella et al. (2018) reported no significant association between OSP and body fat percentage, measured by body scale, among 12-year-olds [36]. Not only dichotomizing OSP but also the method used to estimate adiposity may serve as an explanation for the discrepancy reported. In the current study, adiposity was estimated by a DXA scan although BMI is the more commonly used method. BMI is considered a reasonable measure of adiposity in adolescents, but it may not be a good indicator among sport participants because they tend to be more muscular [37]. This could explain why BMI was not associated with OSP in the current study. The most probable reason for the lower adiposity level among sport participants is the higher physical activity levels, which increase energy expenditure. The association was, however, independent of total physical activity. It can be argued that measuring physical activity in one week might not accurately reflect total physical activity levels over a longer period of time. Energy intake might serve as a possible explanation since engagement in sports has been associated with better overall nutrient intake, which promotes energy balance [38].

### 4.4. Sleep and Screen Time

In the literature, the association between OSP and sleep in inconclusive. The current findings are in line with those of Brand et al. (2010) showing no difference in self-reported sleep duration between participating and non-participating adolescents who engaged in sport [39]. The authors did, however, report favorable sleep patterns (e.g., better sleep quality, lower number of awakenings) among participants who engaged in sport [39]. Driller et al. (2017) reported shorter sleep duration among university athletes measured by accelerometry [40], but Mäkelä et al. (2016) reported longer sleep duration in 14–16-year-olds during weekends using self-reported data [41]. The method used to assess sleep duration may serve as an explanation [42]. Based on the results from objectively measured sleep duration, it can be speculated that the importance of healthy lifestyle habits, including sufficient sleep, are not promoted enough by coaches in sport clubs.

OSP was negatively associated with screen time in the current study with the threshold set at four times a week. These findings are supported by the findings of Allen et al. (2015) who reported a weak negative but significant association between OSP and screen time in 12-year-olds [43]. These findings indicate that screen time may be displaced by time spent in OSP but, even so, participants in the current study were spending close to three times the maximum recommended limit of two hours of screen time. In addition to increasing physical activity levels, OSP may also be promoting adolescents’ health by decreasing screen time, which requires a very low level of physical activity.

### 4.5. Strengths and Limitations

This study possesses several strengths. First, physical activity and sleep duration were objectively measured by an accelerometer worn on the wrist. The use of a wrist accelerometer to assess activity and sleep over the 24 h cycle is less of a burden than a hip accelerometer for young subjects [44]. Sleep detection algorithms have been well validated for the wrist actigraphy [26]. However, there are no known standards yet for interpreting intensity of wrist accelerometer data [45,46], for outcome measures such as MVPA. The accelerometer, our principal assessment tool for physical activity, cannot discriminate between being sedentary and physically active if no movement occurs at the part of the body where the monitor is placed [47].

Second, body composition was estimated by a DXA scan and, third, participants were categorized according to OSP frequency allowing for exploration of a dose dependent association or a threshold effect rarely seen in previous studies.

Limitations include the relatively low number of participants, which limited the possibility of exploring each sex in more depth after the OSP frequency categorization and the cross-sectional design, which makes it difficult to reach a definite conclusion on causal relationships. The use of self-reported screen time can also be viewed as a limitation, especially among girls [48].

Future studies on this topic should consider the type of sport participants are engaged in as level of physical activity varies between different sports. Future studies should also strive to include higher number of participants and stratify participants by sex. Even though we adjusted for sex in all analyses and tested for interaction between sex and study variables, exploring the sex separately will shed light on any sex-based difference in the association between OSP and health related variables. Future studies should include questions regarding smart phone use when exploring screen time.

## 5. Conclusions

OSP is associated with more favorable physical activity levels, CRF, screen time, and body composition. Participating in organized sport four times per week, at minimum, is required for the effect to be significant. The impact of intensive OSP on participants’ health may have been underestimated in previous studies dichotomizing OSP, participants vs. non-participants. The findings reveal that OSP may serve as a valuable tool in battling low physical activity levels among adolescents and encourage its promotion by communities and sport clubs to enhance the overall health and lifestyles of Icelandic adolescents. These findings could be used to inform policies at educational, recreational, and healthcare levels to enhance the overall quality of life and well-being of children and adolescents.

## Figures and Tables

**Table 1:** Participants’ characteristics.

	All			Boys			Girls		
	N	M	SD	N	M	SD	N	M	SD
Age (years)	315	15.85	0.32	132	15.81	0.33	183	15.87	0.32
Height (cm)	306	171.48	8.04	126	178.23	5.72	180	166.76 *	5.74
Weight (kg)	306	64.71	11.18	126	68.96	11.31	180	61.73 *	10.10
Fat%	307	26.39	9.03	127	19.37	7.68	180	31.34 *	6.17
FFM (kg)	303	47.36	8.55	125	55.09	6.62	178	41.93 *	4.70
PA (ctm)	300	2028.42	464.04	121	2010.74	463.20	179	2040.38	465.52
CRF (maxW)	250	182.81	49.32	108	225.4	39.89	142	150.41 *	25.28
CRF (W/kg_FFM_)	248	3.81	0.54	108	4.09	0.54	140	3.60 *	0.43
Screen time (hrs)	299	5.85	2.56	126	6.21	2.54	173	5.59 *	2.58

M = mean, SD = standard deviations, PA = physical activity, Fat% = body fat percentage, FFM = fat free mass, CRF = cardiorespiratory fitness. * *p* < 0.05.

**Table 2:** Linear regression results with organized sport participation (OSP) as the dependent variable.

	N	df	F Value	*p* Value	PES
Behavior					
PA ^a^	294	3, 286	14.53	< 0.01	0.13
-Boys	120	3, 115	13.07	< 0.01	0.25
-Girls	174	3, 171	4.69	< 0.01	0.08
CRF ^b^	242	3, 236	17.64	< 0.01	0.18
Sleep ^c^	294	3, 290	0.37	0.78	< 0.01
Screen time ^c^	296	3, 294	8.14	< 0.01	0.08
Body composition					
Fat% ^c^	297	3, 292	11.84	< 0.01	0.11
FFM ^c^	297	3, 290	5.76	< 0.01	0.06

ANCOVA models adjusted for: ^a^ OSP/sex interaction, ^b^ sex and FFM, ^c^ sex. PA = physical activity, CRF = cardiorespiratory fitness, fat% = body fat percentage, FFM = fat free mass. PES = partial eta squared.

**Table 3:** Groups’ mean comparisons in health behavior and body composition.

	Number of Weekly Practices											
	0 (Group 1)			≤3 (Group 2)			4–5 (Group 3)			6–7 (Group 4)		
	N	Mean	SE	N	Mean	SE	N	Mean	SE	N	Mean	SE
Behavior												
PA (ctm) ^a^	92	1838.4	48.91	53	1875.7	60.73	76	2160.6 ^ƚ^*	51.54	73	2223.3 ^ƚ^*	51.21
-Boys	29	1781.1	76.01	22	1701.4	87.30	29	2241.4 ^ƚ^*	76.04	40	2176.5 ^ƚ^*	64.74
-Girls	63	1895.7	57.05	31	2050.0	81.33	47	2079.7	66.05	33	2270.3 ^ƚ^	78.8
CRF ^b^	76	173.6	2.54	44	176.6	3.28	64	191.5 ^ƚ^*	2.75	58	197.8 ^ƚ^*	2.93
Sleep (min)	92	391.2	4.81	53	398.0	6.74	76	394.8	4.56	73	394.4	4.59
ST (hrs) ^c^	94	6.74	0.26	53	6.40	0.39	78	5.12 ^ƚ^*	0.28	74	5.36 ^ƚ^*	0.29
Body comp												
Fat% ^c^	97	27.5	0.66	53	27.2	0.88	76	24.5 ^ƚ^	0.74	71	22.1 ^ƚ^*	0.76
FFM ^c^ (kg)	97	47.6	0.57	53	46.9	0.75	76	49.4	0.63	71	50.2 ^ƚ^*	0.65

^a^ Adjusted for sex/OSP interaction, ^b^ adjusted for sex and FFM, ^c^ adjusted for sex, ^ƚ^ significantly different from the not-actively engaged group (zero practice sessions per week), * significantly different from the group with three or less practice sessions per week (≤3). PA = physical activity, CRF = cardiorespiratory fitness, ST = screen time, Fat% = body fat percentage, FFM = fat free mass. Estimated means displayed where covariates were included in the analyses.

## Data Availability

Datasets for the current study are available from the corresponding author upon reasonable request.
